# Comment on: Malfitano, A.M. et al. “Virotherapy as a Potential Therapeutic Approach for the Treatment of Aggressive Thyroid Cancer” *Cancers* 2019, *11*, 1532

**DOI:** 10.3390/cancers12020263

**Published:** 2020-01-22

**Authors:** Pēteris Alberts

**Affiliations:** Rigvir, Atlasa iela 7C, LV-1026 Riga, Latvia; peteris.alberts@rigvir.com

I read with interest the recent review on virotherapy in thyroid cancer by Malfitano et al. [1].

For the review, to provide full and correct information of the oncolytic ECHO-7 virus Rigvir, I would like to add the following.

On page 2, paragraph 6, the authors write that “Rigvir, a wildtype non-pathogenic enteric cytopathic human orphan type 7 (ECHO-7), was approved only by the Latvian regulatory agency for the treatment of melanoma”.

Rigvir has in fact so far been approved not only in one, but four, independent countries. Registrations were granted in Latvia in 2004, Georgia in 2015, Armenia in 2016, and Uzbekistan in 2018 [2].

Next, the authors state that “This virus has not met the required standard regulation for European or U.S. approval, further studies are in progress to better evaluate its therapeutic potential”.

This statement appears to assume that the Rigvir dossier would have been submitted for registration to the mentioned regulatory agencies and that it would have been found not to meet the regulations. In order to avoid any misunderstanding, it should be noted that this is not the case. In fact, the Rigvir dossier has not been submitted to the mentioned agencies for registration purposes and, consequently, has not been evaluated. Furthermore, the European Medicines Agency (EMA) is aware of the Rigvir drug development plans and this work is continuing.
